# Integrated Analysis Reveals together miR-182, miR-200c and miR-221 Can Help in the Diagnosis of Prostate Cancer

**DOI:** 10.1371/journal.pone.0140862

**Published:** 2015-10-20

**Authors:** Yinmin Gu, Danqing Lei, Xia Qin, Panyu Chen, Yi ming Zou, Yanling Hu

**Affiliations:** 1 Experimental Center of Medical Sciences, Guangxi Medical University, Nanning, Guangxi, China; 2 Dermatological Department, First Affiliated Hospital of Guangxi Medical University, Nanning, Guangxi, China; 3 Center for Genomic and Personalized Medicine, Guangxi Medical University, Nanning, Guangxi, China; 4 Department of Mathematical Sciences, University of Wisconsin-Milwaukee, Milwaukee, Wisconsin, United States of America; UCSF / VA Medical Center, UNITED STATES

## Abstract

Research has shown that microRNAs are promising biomarkers that can be used to promote a more accurate diagnosis of cancer. In this study, we developed an integrated multi-step selection process to analyze available high-throughput datasets to obtain information on microRNAs as cancer biomarkers. Applying this approach to the microRNA expression profiles of prostate cancer and the datasets in The Cancer Genome Atlas Data Portal, we identified miRNA-182, miRNA-200c and miRNA-221 as possible biomarkers for prostate cancer. The associations between the expressions of these three microRNAs with clinical parameters as well as their diagnostic capability were studied. Several online databases were used to predict the target genes of these three microRNAs, and the results were confirmed by significant statistical correlations. Comparing with the other 18 types of cancers listed in The Cancer Genome Atlas Data Portal, we found that the combination of both miRNA-182 and miRNA-200c being up-regulated and miRNA-221 being down-regulated only happens in prostate cancer. This provides a unique biological characteristic for prostate cancer that can potentially be used for diagnosis based on tissue testing. In addition, our study also revealed that these three microRNAs are associated with the pathological status of prostate cancer.

## Introduction

Prostate cancer (PCa) is the second most frequently diagnosed cancer and is the sixth highest cause of cancer-related death among men worldwide [1]. It is a clinically heterogeneous-multifocal disease, and the number of cases is steadily increasing [2]. So far, prostate-specific antigen (PSA) detection has provided the most effective biomarker for diagnosis and the response to treatment in PCa. However, the sensitivity and specificity of PSA testing are insufficient, which results in low detection rates [3]. With the advancement of research on carcinogenesis, PCa studies have increasingly focused on new strategies for early detection and prevention [4].

Studies have suggested that microRNA (miRNA), a type of endogenous, small, non-coding RNA with an approximate length of 22 nucleotides [5], may be linked to cancer; specifically, aberrant miRNAs are linked to clinical behavior and they can be promising biomarkers for more accurate diagnostic/prognosis of cancers [6,7,8]. Using miRNAs as potential diagnostic markers in PCa has been reported in the literature. It has been reported that miR-141 is elevated in the serum of PCa patients and correlates significantly with PSA [9]. Moreover, it has been shown that a five-miRNA panel (downregulation of let-7e, let-7c and miR-30c, upregulation of miR-622 and miR-1285) is capable of accurately differentiating PCa from benign prostate hyperplasia (BPH) and normal samples [10]. These reports suggested that identifying aberrations in miRNAs associated with a particular type of cancer should provide good biomarkers for these specific cancers and promote earlier diagnosis.

Today, high-throughput technologies have produced a large amount of cancer data, so it is desirable to use these data to identify miRNAs and the aberrations that are associated with different types of cancers. However, the analyses of these high-throughput data face difficulties. One difficulty is the lack of homogeneity among different sets of miRNA data due to the fact that different platforms were used to acquire them. Different sets of miRNA data that are expressed differently tend to show inconsistencies with each other. Among the methods developed to address this problem, the robust rank aggregation (RRA) method, which defines the rank vector for each gene based only on the datasets where it is present, has been shown to provide statistically significant miRNA meta-signatures [11, 12]. The method is based on using order statistics to compare each gene to the baseline case, where all the preference lists are randomly shuffled, and then assigns significance levels to the findings [11]. However, in order to accurately identify the potentially useful miRNAs as biomarkers from the selected miRNAs using RRA, further statistical analysis and verification are necessary in addition to the RRA method.

In this study, we applied a new multi-step selection approach to the existing high-throughput data of PCa for the purpose of identifying miRNAs as PCa biomarkers. We first applied the RRA method to select potential miRNA biomarkers in prostate tumors using 11 published miRNA expression profiles. Then, we used The Cancer Genome Data Atlas (TCGA) to further verify the selected miRNAs in multiple ways by the Wilcoxon rank sum test. We found that the combination of two up-regulated miRNAs (miRNA-182, miRNA-200c) and one down-regulated miRNA (miRNA-221) is unique in PCa. This suggests that this combination of miRNAs and their expression levels could potentially provide additional effective diagnostic indicators for PCa.

## Materials and Methods

### Literature search

The information on miRNA expression profiling studies on PCa was systematically searched in PubMed, Embase and Highwire databases, using the search string (prostate and (cancer* OR tumor* OR tumour*) and (mirna* OR microrna * OR mir-*)). In addition, we obtained miRNA expression profiles for PCa through searching the Gene Expression Omnibus (GEO) (http://www.ncbi.nlm.nih.gov/geo/) [13] and ArrayExpress (http://www.ebi.ac.uk/arrayexpress/) repositories [14]. The search was restricted to data published between January 1, 2005 and January 31, 2014. Our selection criteria were: (a) original experimental articles providing a comparison of prostate tumor tissues and non-tumor tissues, (b) studies were about miRNA expressions, (c) the studied organism was *Homo sapiens* and (d) viral miRNAs and non-miRNA probes were excluded.

In this study, the non-tumor tissues included prostate tissues adjacent to a tumor and tissues from independent healthy donors, but did not include BPH. The data extracted from each study included: first author, Gleason score, region, assay type, the number of miRNA probes and the number of samples. The lists of miRNAs with statistically significant expressions were either extracted from the publications or obtained from the authors directly.

### Accessing and processing of TCGA data

The data including normalized miRNA-HiSeq expression values, raw read counts of mRNA-seq and clinical information for PCa were downloaded by the “TCGA-Assembler” package in R [15]. Level 3 HiSeq genes normalized data for PCa were acquired from FIREHOSE Broad GDAC (http://gdac.broadinstitute.org/). Reads per kilobase of exon model per million mapped (RPKM) normalized values for mRNA expressions and reads per million miRNA mapped (RPM) normalized values for miRNA expressions were further log_2_-transformed. Fold changes in miRNA expressions between tumors and normal tissues were calculated using median-centered RPM values. Based on raw read counts, differential mRNA expression analysis was performed by the DESeq Bioconductor package in R [16], which uses a negative binomial distribution model and local regression to estimate the relationship between the mean and variance of each gene. All differentially expressed genes were considered significant if the absolute values of their log_2_ fold changes were greater than 1 and the false discovery rates (FDR) were < 0.1.

### Prediction of the target genes of miRNA and enrichment analysis

Target gene predictions of differentially expressed miRNAs were performed using TargetScan [17], miRwalk [18] and the PICTAR database [19]. The predicted targets must have been selected by at least two algorithms. Validated targets from the CLIP-Seq database starBase [20] were also used in our selection process. The selected target genes were the overlapping targets of the predicted targets and the validated target genes. The target genes in the following discussions show significant correlations with the expressions of miRNAs. The DAVID tool [21] was used to elucidate the molecular functions of the candidate miRNAs.

### Statistical analysis

The RRA method we used was adopted from a previous study [12]. We integrated 11 miRNA lists to make sure that the lists were ranked consistently better than expected by the RobustRankAggreg package in R [11] using the function “aggregateRanks”. The extracted miRNA lists were first prioritized based on statistical test *p*-values (less than 0.05 was considered significant). If the *p*-value was not reported, we used the fold change (FC) instead. Leave-one-out cross-validation (LOOCV) was performed after the RRA analysis to assess the stability of the acquired *p*-values. We found that the *p*-values stabilized after 10,000 runs of this analysis. We thus used 10,000 tests as the cutoff, excluded one random miRNA list for each test, and used the average *p*-value of each miRNA as the final *p*-value.

Then, the Spearman’s rank correlation coefficients and the two-tailed *p*-values of the miRNAs were estimated using the “cor.test” function in R. The relationships among clinical features and the expressions of the miRNAs were evaluated by Wilcoxon rank sum or Kruskal-Wallis non-parametric test using the “wilcox.test” function or the “kruskal.test” function in R, respectively. The diagnostic capabilities of miRNAs were assessed using the “pROC” package in R. All statistical calculations were performed on log_2_ transformed expression levels.

## Results

### Selection of PCa meta-signature miRNA candidates

**Fig 1:**
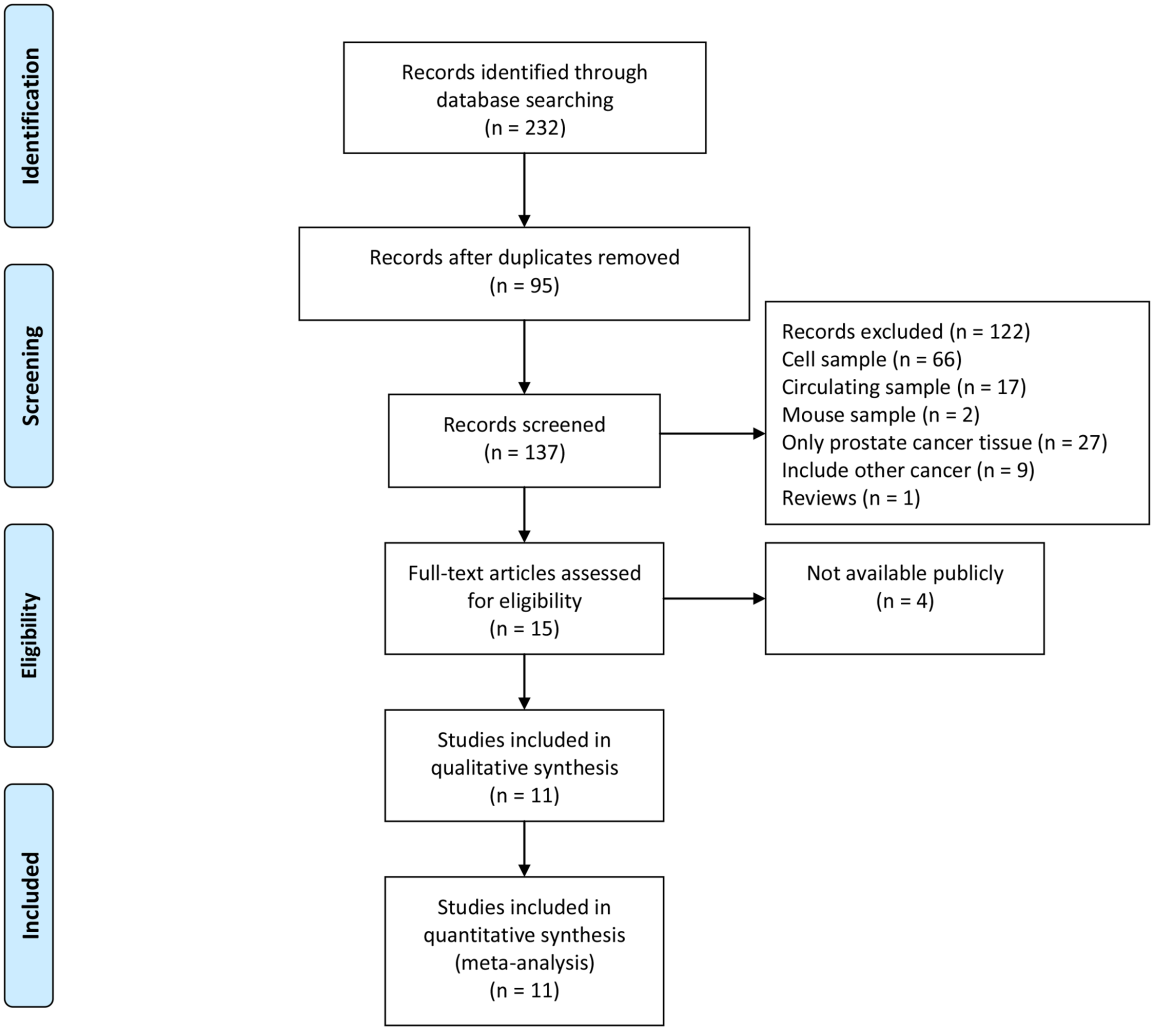
Searching strategy.

Our selection criteria (see Materials and Methods) resulted in the selection of 11 miRNA profiling datasets for our analysis (Fig 1). Six of the studies also provided Gleason scores or carcinoma tissues, so we classified them as the carcinoma group. A brief overview of the 11 selected datasets is presented in Table 1. In total, we analyzed 347 carcinoma samples and 188 normal samples. These datasets included various microarray platforms and the number of miRNA probes ranged from 88 to 847. The results from RRA provided eight statistically significant miRNAs consisting of four up-regulated and four down-regulated markers. After the stability of the *p*-value was tested, miR-375 (*p* = 0.053 > 0.050) and miR-25-3p (*p* = 0.074 > 0.050) were excluded, and the other six meta-signature miRNAs (*p* < 0.050) were kept for further analysis. These six selected miRNAs included two up-regulated miRNAs (miR-182-5p, miR-200c-3p) and four down-regulated miRNAs (miR-145-5p, miR-205-5p, miR-221-3p, and miR-222-3p).

**Table 1:** Brief overview of the datasets.

First author and reference	Gleason score	Region	Assay type	Number ofmiRNA probes	Number ofsamples
Stefan Ambs [22]	5(2); 6(18); 7(54);8:(1)	North America	OSU-CCC hsa-miRNA-chip version 3	329	60 TU+16 N
Pei-Chun Lin [23]	NA	North America	Affymetrix GeneChip array	847	21 Pairs
Sven Wach [24]	7(18); 9(2)	North America	Affymetrix GeneChip array	847	20 Pairs
Annika Schaefer [25]	NA	Europe	Agilent Human miRNA Microarray	470	12 Pairs
ES Martens-Uzunova [26]	6(35); 7(15); 8(13);9(2)	Europe	Agilent Human miRNA v2 Microarray	723	72 TU(1)+15 N
Jessica Carlsson [27]	6(6); 7(7); 8(1); 9(4);10(2)	Europe	qPCR	667	20 Pairs
Jessica Carlsson [28]	3(13)	Europe	qPCR	667	13 TU+10 N
Stefano Volinia [29]	NA	North America	Custom microarray	2285	56 TU+7 N
AW Tong [30]	NA	North America	mirMASA technique	114	20 TU+20 N
M Ozen [31]	NA	North America	Custom microarray	480	16 TU+10 N
A Bertriz [32]	6(5); 7(21); 8(10); 9(4)	North America	qPCR	88	37 Pairs

Abbreviations: TU, tumor sample; N, non-tumor sample; NA, not available; in the last column, the pairs, TU and N samples were from the same patient. TU(1): 72 tumor samples included 50 organ-confined PCa samples and 22 malignant trans-urethral resection of the PCa (TURP-PCa) samples. The numbers 5(2) indicate that the Gleason score was 5 for 2 PCa cases, the same as for the others.

### Validation of meta-signature miRNAs using TCGA database

**Fig 2:**
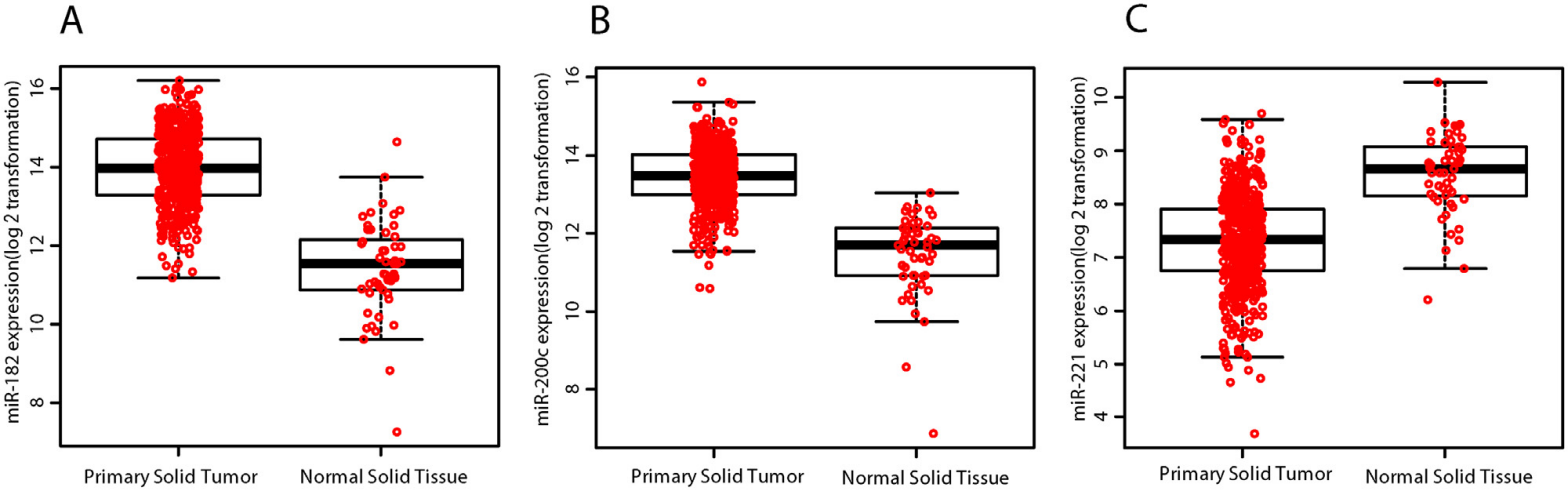
TCGA data for the three differentially expressed miRNAs in prostate primary solid tumor (n = 498) and normal solid tissue (n = 52). (A) The expression levels of miR-182 in prostate primary solid tumor and normal solid tissue. (B) The expression levels of miR-200c in prostate primary solid tumor and normal solid tissue. (C) The expression levels of miR-145 in prostate primary solid tumor and normal solid tissue. The expression level values were calculated using log_**2**_ transformed RPM values.

**Fig 3:**
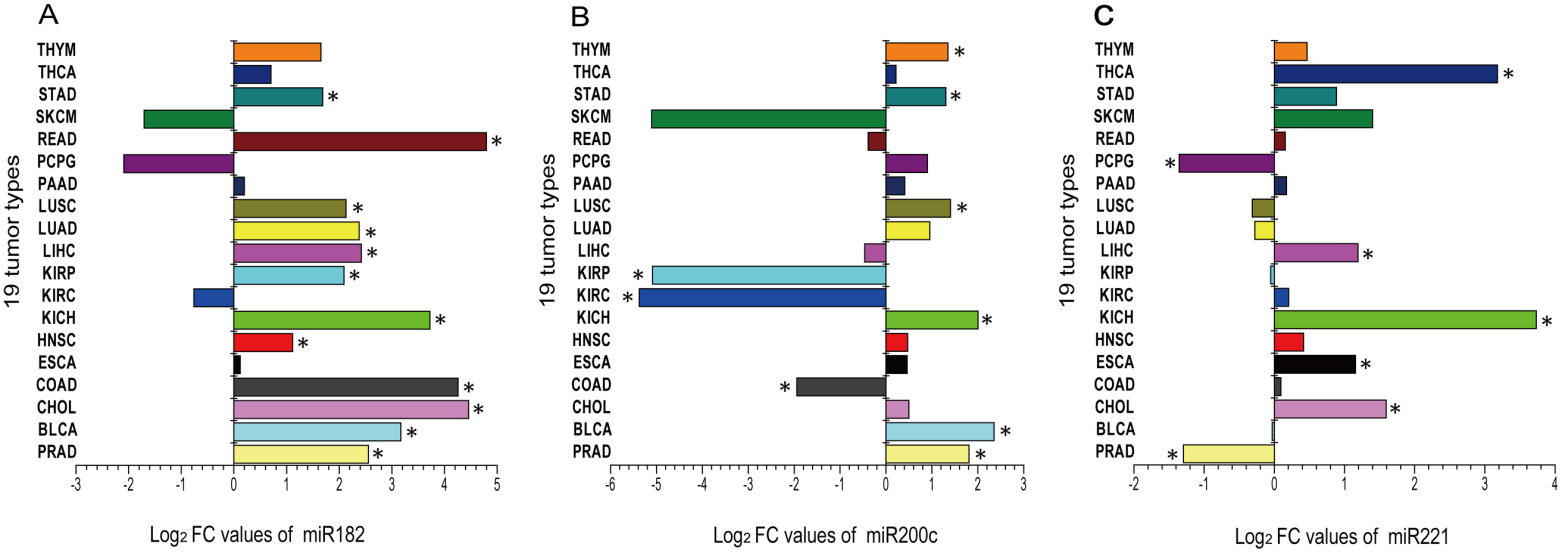
The log_2_ FC values of the three miRNAs in 19 cancer types. Fold change is the ratio of the median signals between cancer tissue and normal tissue. Abbreviations: FC, Fold change; PRAD, Prostate adenocarcinoma; BLCA, Bladder urothelial carcinoma; CHOL, Cholangiocarcinoma; COAD, Colon adenocarcinoma; ESCA, Esophageal carcinoma; HNSC, Head and neck squamous cell carcinoma; KICH, Kidney chromophobe; KIRC, Kidney renal clear cell carcinoma; KIRP, Kidney renal papillary cell carcinoma; LIHC, Liver hepatocellular carcinoma; LUAD, Lung adenocarcinoma; LUSC, Lung squamous cell carcinoma; PCPG, Pheochromocytoma and paraganglioma; READ, Rectal adenocarcinoma; STAD, Stomach adenocarcinoma; THCA, Thyroid carcinoma; THYM, Thymoma; PAAD, Pancreatic adenocarcinoma; and SKCM, Skin cutaneous melanoma. * The indicated miRNA expressed differentially in a cancer.

TCGA database was used to validate the expression level changes of the six selected meta-signature miRNAs in PCa patients. In the validation, the corrected *p* -value cut-off was set to 0.05 and the FC cut-off was set to 2 in order to provide more rigorous identification indicators to differentiate miRNAs. Based on the analysis of 498 PCa samples and 52 normal samples, we found that three statistically significant miRNAs were in agreement with the analysis result of the expression profiling by RRA (Table 2 and Fig 2). Specifically, miR-182 (FDR = 3.50E-26, FC = 5.89) and miR-200c (FDR = 1.10E-26, FC = 3.50) were significantly up-regulated, miR-221 (FDR = 1.43E-16, FC = 0.41) was significantly down-regulated, and the FC of miR-145, miR-205 and miR-222 were greater than 0.5 (FDR = 3.29E-01, 6.50E-06 and 1.33E-12, respectively; FC = 0.90, 0.52 and 0.52, respectively). To further verify our selection of these three miRNAs as potential biomarkers for PCa, we then investigated their expression levels in 18 other tumor types in TCGA data. The analytical results from these three miRNAs in 18 other tumor types and PCa are shown in S1 Table and Fig 3, respectively. These results showed that the event of up-expression of miR-182 and miR-200c and down-expression of miR-221 can only be observed in PCa.

**Table 2:** Candidate miRNAs for PCa biomarkers.

miRNA	Chromosome	Corrected*p*-value	Permutation*p*-value	Total number of studies	Seed family
miR-182-5p	7q32.2	4.52E-04	5.43E-05	5	miR-182
miR-200c-3p	12p13.31	4.54E-02	1.06E-02	5	miR-200a/miR-200b/miR-200c/ miR-429/ miR-141
miR-221-3p	Xp11.3	2.19E-04	2.03E-05	7	miR-221/222/222ab/1928

Next, we used the Spearman rank correlation to test the association among the three identified miRNAs in PCa. We found that miR-182 and miR-200c showed a positive correlation (*r*_*s*_ = 0.633, *p* < 2.200E-16), and they were negatively correlated with down-regulated miR-221 (miR-182 vs miR-221: *r*_*s*_ = -0.479, *p* < 2.200E-16; miR-200 vs miR-221: *r*_*s*_ = -0.335, *p* = 1.983E-14). Therefore, we carried out further analysis to confirm these three miRNAs and their unique combination of expression levels in PCa.

To explore the expression levels of miR-182, miR-200c and miR-221 in PCa blood or serum, we used GEO2R [33] to analyze the data, we found that miR-182 (log_2_ FC = 1.740, *p* = 0.046) and miR-200c (log_2_ FC = 1.963, *p* = 0.009) showed overexpressions and miR-221 (log_2_ FC = -0.810, *p* = 0.110) showed no significant difference in the series GSE24201 [34], which included 14 blood samples of PCa patients and 15 samples of healthy brothers from 11 families. We also compared the serums of 3 TRansgenic Adenocarcinoma of Mouse Prostate (TRAMP) form mice to their 3 wild-type littermates from GSE29314 [35], and we found that miR-182 (log_2_ FC = 1.187, *p* = 0.010) and miR-200c (log_2_ FC = 1.389, *p* = 0.002) were also up-regulated and reached *p* < 0.05, while miR-221 (log_2_ FC = -0.047, *p* = 0.818) did not show a different trend.

### Correlation of the expressions of the three selected miRNAs with clinicopathologic parameters

The clinicopathologic features of the PCa patients obtained from TCGA are shown in S2 Table. Their mean age was 60.4±7.0 and their pre-surgery PSA ranged 0.7–87 (lg/l). Our Spearman rank analysis showed that these three miRNAs were significantly associated with pre-surgery PSA levels and ages. Both miR-182 and miR-200c showed a positive correlation with the two clinical parameters while miR-221 showed a negative correlation. These three miRNAs did not show significant differences among races (Table 3). We found that miR-221 showed significant differentiation in terms of Gleason grade (low Gleason score vs. high Gleason score), lymph node positivity, the pathology of the N stage (pN0 vs. pN1), and the pathology of the T stage (pT2 vs. pT3 vs. pT4) (*p* < 0.05 for all comparisons) (see Table 3), and it was down-regulated in patients with higher Gleason scores, advanced stage tumors and positive lymph nodes. However, we did not find significant differences between these clinical features and miR-182 or miR-200c.

**Table 3:** Correlations between the expressions of the three miRNAs and clinical features.

Characteristic	miR-182	miR-200c	miR-221
Age	*p* = 0.006*r*_*s*_ = 0.170	*p* = 0.005*r*_*s*_ = 0.177	*p* = 0.038*r*_*s*_ = -0.129
Race	*p* = 0.358	*p* = 0.287	*p* = 0.905
Pre-surgery PSA Levels	*p* = 0.005 *r*_*s*_ = 0.173	*p* = 0.036 *r*_*s*_ = 0.131	*p* = 0.001*r*_*s*_ = -0.210
Low Gleason Score (6–7) vs High Gleason Score (>8)	*p* = 0.105	*p* = 0.355	*p* = 3.069E-06
Number Of Positive Nodes	*p* = 0.559*r*_*s*_ = 0.128	*p* = 0.332*r*_*s*_ = 0.212	*p* = 0.774*r*_*s*_ = 0.063
Positive Node vs Negative Node	*p* = 0.059	*p* = 0.561	*p* = 0.002
Positive Node (1–2) vs 2 more	*p* = 0.428	*p* = 0.325	*p* = 0.776
Pathology N Stage (pN0 vs pN1)	*p* = 0.054	*p* = 0.604	*p* = 0.001
Pathology T Stage (T2 vs T3 vs T4)	*p* = 0.204	*P* = 0.784	*p* = 0.003E-01
Histological Type	*p* = 0.003	*p* = 0.017	*p* = 0.115

Abbreviation: *r*_*s*_, Spearman’s rank correlation coefficient

### Diagnostic value of the selected miRNAs

**Fig 4:**
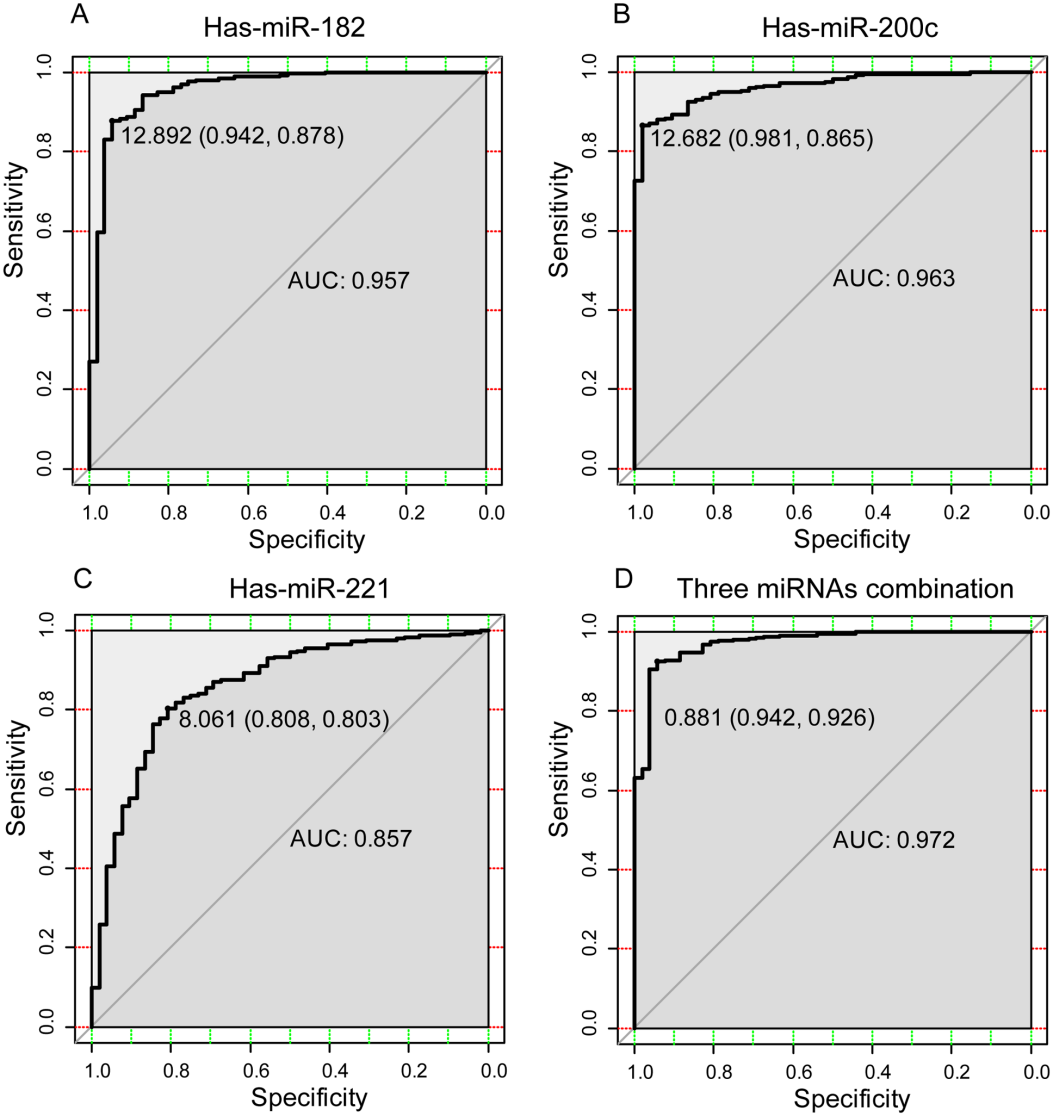
The areas under the ROC curves for the three identified miRNAs and their combination that differentiate between PCa samples and normal samples. (A) The ROC curve for miR-182. (B) The ROC curve for miR-200c. (C) The ROC curve for miR-221. (D) The ROC curve for the combination of the three miRNAs.

We conducted response operating characteristic (ROC) analyses to evaluate the use of the three selected miRNAs as potential biomarkers to differentiate PCa tissues from normal tissues (Fig 4), and we found that MiR-200c (AUC = 0.963, 95% confidence interval, CI = 0.9463–0.980, *p* < 0.0001) showed a higher diagnostic capability than miR-182 (AUC = 0.957, 95% confidence interval, CI = 0.9248–0.9896, *p* < 0.0001) and miR-221 (AUC = 0.857, 95% confidence interval, CI = 0.8022–0.9127, *p* < 0.0001). When a logistic regression approach was used for the combination of these three miRNAs, the ROC curve revealed a much better diagnostic accuracy than if they were used individually, the results showed an AUC of 0.972 (95% confidence interval, CI = 0.9519–0.992, *p* < 0.0001). In the analysis, the optimal cutoff value was set at a maximal sum of sensitivity and specificity. The diagnostic sensitivity and specificity of the combination of these three miRNAs were found to be 94.2% and 92.6%, respectively.

### Target genes and biological pathway recognition

**Fig 5:**
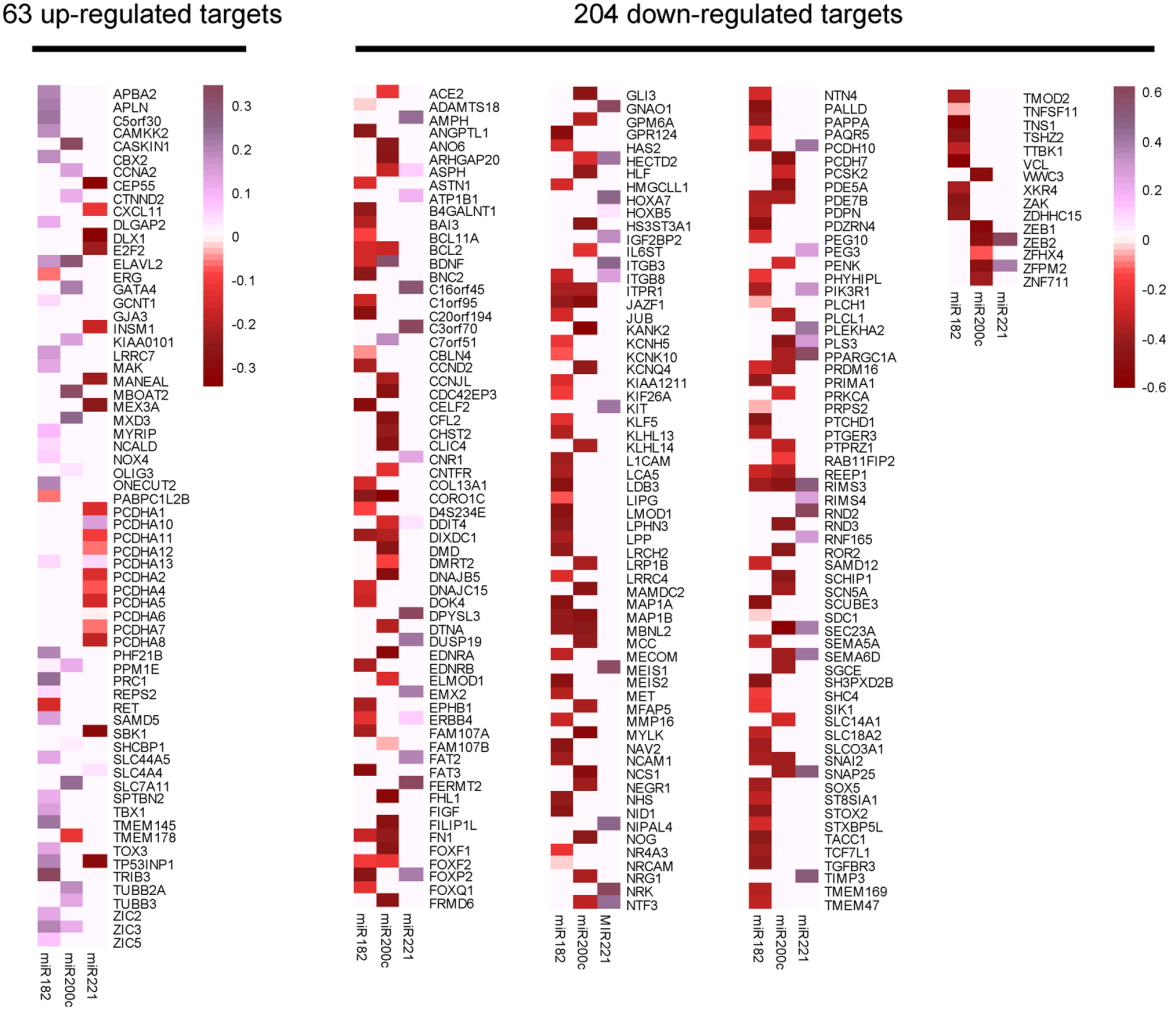
Clusters of miRNA-target genes by their correlation coefficients. The heat map representation of the correlation patterns for the miRNA targets. The 267 differentially expressed target mRNAs, including 63 up-regulated targets and 204 down-regulated targets, were used to cluster the miRNAs. Rows of the heat map represent target mRNAs, and columns represent the miRNAs. Red squares indicate negative correlations, purple squares indicate positive correlations and white spots (colorless) indicate that there is no correlation.

First, we identified 800 up-regulated and 1698 down-regulated different genes based on a model using the negative binomial distribution for 374 prostate cancers samples and 52 normal controls from TCGA (S3 Table). Second, we predicted target genes for these three miRNAs, and then chose the overlap genes as pathway participants (129 genes for miR-182, 95 genes for miR-200c, and 55 genes for miR-221) (S4 Table). Third, we performed correlation analysis among these three miRNAs and the mRNA expressions of all overlap genes. The results showed that miR-182 and miR-200c were positively associated with almost all up-regulated overlap mRNAs and negatively associated with almost all down-regulated mRNAs. However, the correlation between miR-221 and the overlap genes showed an inverse relation (Fig 5). A surprising observation from our analysis was that regulating synaptic membrane exocytosis 3 (*RIMS3*) consistently overrepresented with all three miRNAs.

**Fig 6:**
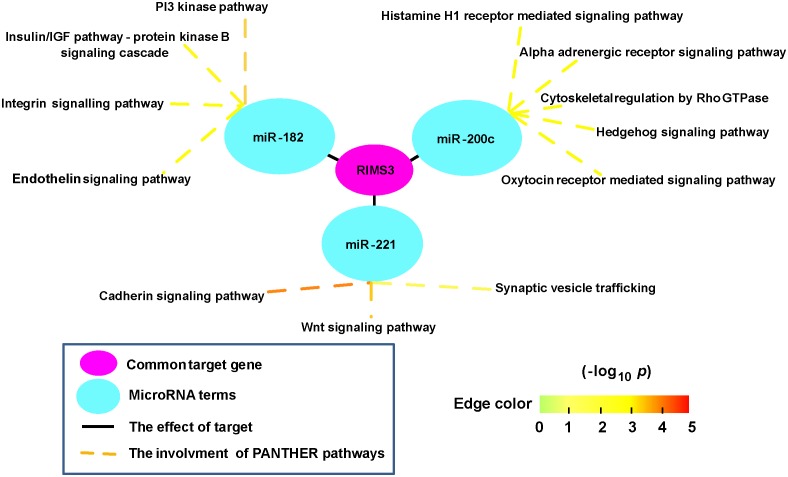
Common target genes and biological pathways of the three miRNAs. The purple oval represents *RIMS3*; the three blue ovals represent the three miRNAs; black straight lines indicate the target effect of miRNAs on gene; dotted lines indicate the involvement of the miRNAs in PANTHER pathways, and their colors reflect the significances of -log_10_ transformed *p* values for the pathways.

To understand the general biological functions of these three miRNAs, we conducted Protein Analysis Through Evolutionary Relationships (PANTHER) pathway analysis in DAVID via the target genes. The results showed that these three miRNAs did not share the same pathways, but they were frequently related to cell signaling pathways (Fig 6).

## Discussion

In recent years, many studies have been devoted to the discovery of miRNA biomarkers and their biological functions (or molecular mechanism) for PCa. In this study, we applied an integrated multi-step selection approach to analyze the cancer datasets from GEO and TCGA and identified three miRNAs and their unique expression combination pattern that only showed in the PCa datasets.

The notion that miRNAs are implicated in cancer is supported by the evidence that miRNAs are located largely in genomic regions associated with cancer or at fragile sites [36, 37]. For PCa, we can obtain the following information from the literature. It is known that miR-200c is located at 12p13.31, and it was reported [38] that the copy numbers of chromosome region 12p13.31-p12.3 are deleted in PCa. It has also been reported [39] that chromosome region 7q32.2 in which miR-182 sits has a high incidence of heterozygosity and/or microsatellite imbalance alterations in prostate carcinogenesis. Histone methylation results in the silencing of the entire miR-221/miR-222 cluster, as clarified by an analysis of the methylation signature in PCa cell lines [40]. Therefore, just based on the published information on these locations, we may form the hypothesis that miR-182, miR-200c and miR-221/miR-222 are closely related to PCa.

It was also reported that miRNA-182 is over-expressed in PCa and plays an active role in proliferation and invasion in *vitro* and *vivo* [41, 42]. It has been reported that miR-182 in PCa tissues and four cell lines (LNCap, PC-3, DU145, and 22Rv1) have higher levels than in BPH tissues and normal prostatic epithelial (RWPE-1) cells [41]. Associated with PCa progression, the over-expression of miR-182 represses the expression of the tumor suppressor gene *FOXF2*, which decreases PCa cell invasion and migration [42]. In prostate cells, miR-182 induces mesenchymal to epithelial transition features and growth factor-independent growth by repressing *SNAI2*, which has been demonstrated to be a repressor of proliferation in PCa cells [43, 44]. Our target gene prediction study correctly identified *FOXF2* and *SNAI2*. The miRNA-200 family, consisting of five members (miR-200a, miR-200b, miR-200c, miR-429, and miR-141), is treated as a tumor suppressor as it inhibits epithelial-to-mesenchymal transition, tumor cell invasion and metastasis [45]. The miR-200c~141 cluster depresses the proliferation of human metastatic prostate cancer cells by inhibiting *JAGGED1*, which may be important for metastases [46]. It has been reported [47] that miR-221 was down-regulated in aggressive PCa and associated with the Gleason score, and thus indicated the tumor stage. Down-regulation of miR-221 has also been detected in prostate secretion samples [48]. In addition, miR-221s are thought to be involved in the development or stability of the castration-resistant prostate cancer (CRPC) phenotype because over-expression has been observed in CRPC cells [49]. Also, many repeated validations have indicated that miR-221 in PCa cells has the ability to regulate the expression of *p27/kip1* and inhibit several cyclin-dependent kinase complexes [50, 51]. All of these are in accordance with our analysis which showed that the three identified microRNA were involved in the development of PCa.

Increasing in age is correlated with PCa risk [52, 53], since levels of miRNAs change with ageing [54], and PCa diagnosis and treatments have been guided by PSA [55]. Our results showed that age and PSA are positively correlated with up-regulated miRNAs (miR-182 and miR-200c), but negatively related to down-regulated miRNA (miR-221). The combination of multi-biomolecules has been proposed to serve as efficient indicators for diagnosis in many studies [56, 57]. Our results showed that the AUC of the combination of these selected three miRNAs was very high for PCa tissues.

Since tumor cells can release miRNAs into the body’s circulation [58], it is desirable to use miRNAs in body fluids for diagnostic purposes if possible. It has been reported that the levels of miRNA in PCa tissue are highly correlated with pre-prostatectomy levels in serum [54]. However, though the combination of increases in miR-182 and miR-200c expressions and the reduction in miR-221 expression is unique (with statistical significance) in PCa blood, further studies will be needed to differentiate these blood markers in PCa from those in LUSC (Lung squamous cell carcinoma) or in BLCA (Bladder urothelial carcinoma) (see Fig 3 and S1 Table).

Through our analyses of target genes and correlations, we found that down-regulated *RIMS3* was present simultaneously with the three identified miRNAs. This has not been reported for PCa before. Also some of the pathways associated with PCa in which the three identified miRNAs participate have been reported before. For examples: (1) It has been reported that, together with *TMPRSS2-ERG*, the PI3-kinase pathway activates prostate oncogenesis [59]; (2) Wnt signaling and its key component β-catenin contribute to prostate tumorigenesis [60]; and (3) the hedgehog signaling pathway is involved in PCa development and in the progression to more aggressive and even therapy-resistant disease states [61]. All of these studies provide additional supporting evidence for our findings.

## Conclusion

To the best of our knowledge, this study is the first to identify and analyze combinations of multiple miRNAs as a potential biomarker for PCa based on the analysis of miRNA microarray and TCGA datasets. Our study shows that an integrated analysis method based on RRA and followed by other statistical analyses and verifications can effectively identify combinations of multiple miRNAs as potential cancer biomarkers. Our results suggest that the three selected miRNAs and their uniquely combined expression pattern in PCa could be of clinical value in addition to existing testing methods. Finally, although we only applied the proposed multi-step selection approach to study high-throughput datasets for the purpose of identifying potential biomarkers for PCa, we believe that this approach can also be applied to investigate other cancer types using similar high-throughput datasets.

## Supporting Information

S1 PRISMA Checklist PRISMA Checklist. (DOC)

S1 Table The expression levels of miR-182, miR-200c and miR-221 in PCa and other 18 cancer types. (XLSX)

S2 Table The demographic information of the PCa patients in the datasets of TCGA. (DOCX)

S3 Table The 800 up-regulated and the 1698 down-regulated different genes identified in PCa samples and normal samples. (XLSX)

S4 Table The result of using Spearman rank correlation to test the association between expression levels of miRNA-182, miR-200c and miR-221 and their predicted targets in PCa patients (n = 481). (XLSX)
