# Supplementary material for: Integrated Analysis Reveals together miR-182, miR-200c and miR-221 Can Help in the Diagnosis of Prostate Cancer
Source: PLoS One. 2015 Oct 20;10(10):e0140862. doi: 10.1371/journal.pone.0140862 (PMC4618846; doi:10.1371/journal.pone.0140862)
Supplement: S2 Table — (DOCX) [file pone.0140862.s003.docx]

**S2 Table. The demographic information of the PCa patients in the datasets of TCGA.**

| **Characteristic** | **Content** | **All case N=498(257)** | **Missing** |
| --- | --- | --- | --- |
| **Age(years)** | Range | 43-77 | 0 |
|  | Median | 61 |  |
|  | Mean (sd) | 60.4(7.0) |  |
| **Race** | WHITE | 218 | 9 |
|  | BLACK OR AFRICAN AMERICAN | 25 |  |
|  | ASIAN | 5 |  |
| **Preoperative PSA (lg/l)** | Range | 0.7-87 | 0 |
|  | Median | 7.3 |  |
| **Gleason score** | 6 | 23 | 0 |
|  | 7 | 169 |  |
|  | 8 | 34 |  |
|  | 9 | 30 |  |
|  | 10 | 1 |  |
| **T stage** | pT2a | 10 | 2 |
|  | pT2b | 3 |  |
|  | pT2c | 108 |  |
|  | pT3a | 86 |  |
|  | pT3b | 44 |  |
|  | T4 | 4 |  |
| **M stage** | MO | 211 | 46 |
|  | M1 | 0 |  |
| **N stage** | pN0 | 192 | 45 |
|  | pN1 | 20 |  |
| **Residual tumor** | R0 | 184 | - |
|  | R1 | 52 |  |
|  | R2 | 3 |  |
|  | Rx | 5 |  |
| **Histological type** | Prostate Adenocarcinoma Acinar Type | 252 | 0 |
|  | Prostate Adenocarcinoma Other Subtype | 2 |  |
| **Number of lymph nodes** | Range | 1-65 | 46 |
|  | Meidan | 8 |  |
| **Lymph node positive** | Positive | 23 | 47 |
|  | Negative | 187 |  |
